# Elucidating the differential antiviral action of a plant growth promoting rhizobacterium against three genetically distant virus species

**DOI:** 10.3389/fpls.2026.1778459

**Published:** 2026-03-13

**Authors:** Despoina Beris, Konstantinos Kotsaridis, Chrysoula Orfanidou, Anastasia Dimopoulou, Christina Varveri, Nikon Vassilakos, Varvara I. Maliogka

**Affiliations:** 1 Laboratory of Virology, Scientific Directorate of Phytopathology, Benaki Phytopathological Institute, Athens, Greece; 2 Plant Pathology Laboratory, School of Agriculture, Faculty of Agriculture, Forestry and Natural Environment, Aristotle University of Thessaloniki, Thessaloniki, Greece

**Keywords:** DNA viruses, induced systemic resistance (ISR), plant growth promoting microorganisms, RNA silencing, RNA viruses, antiviral properties

## Abstract

Viruses pose a major threat to global agriculture, leading to substantial economic losses, whereas effective control measures remain limited. Plant Growth-Promoting Microorganisms (PGPMs) are used as an environmentally friendly approach against bacterial and fungal diseases. Although accumulating evidence show their antiviral potential their use remains limited mainly due to their variable efficacy against distinct viruses. In a previous study, we reported the differential antiviral effect of *Bacillus amyloliquefaciens* strain MBI600 (Serifel^®^, BASF) in tomato plants against tomato spotted wilt virus (TSWV, *Orthotospovirus tomatomaculae*) and potato virus Y (PVY, *Potyvirus yituberosi*). In this study, we aimed to elucidate the molecular basis of the differential response recorded in the antiviral efficacy of MBI600 against genetically distinct virus species. To this end, the antiviral potential of MBI600 was examined against two more important viruses of tomato cultivation, tomato yellow leaf curl virus (TYLCV, *Begomovirus coheni*), and cucumber mosaic virus (CMV, *Cucumovirus CMV*). MBI600 exhibited strong antiviral activity against TYLCV but not against CMV. The transcriptomic analysis of plants treated with MBI600 in the absence and presence of TSWV, TYLCV and CMV, revealed systemic host responses associated with the effectiveness level of the PGPM’s antiviral ability. Our results provide a better understanding of the selective nature of PGPM-mediated antiviral resistance and support PGPMs usage as an effective strategy against viral diseases.

## 1 Introduction

Plant viruses are a significant threat to the agri-food sector worldwide as 50% of the emerging epidemics are of viral etiology with annual losses exceeding 30 billion USD (Nicaise, 2014). Particularly in high-value crops, such as tomato (*Solanum lycopersicum* L.), viruses can cause severe yield losses, that under certain environmental circumstances, can affect 100% of crop production. In the Mediterranean basin, tomato spotted wilt virus (TSWV, *Orthotospovirus tomatomaculae*), tomato yellow leaf curl virus (TYLCV, *Begomovirus coheni*), and cucumber mosaic virus (CMV, *Cucumovirus CMV*) are enlisted among the most economically important viruses that can impact tomato production (Panno et al., 2021). The current management strategies mainly rely on prophylaxis through various means such as the control of the insect vectors with agrochemicals for the cases of persistent viruses (TSWV, TYLCV) and, when possible, the use of resistant cultivars. However, these strategies are often compromised due to environmental constraints, rapid viral genetic evolution and the development of insect resistance to insecticides. As a result, there is an increasing necessity to obtain effective and environmentally friendly means against plant viral diseases.

Plant growth–promoting microorganisms (PGPMs) are well-known not only to enhance plant growth but also to activate plant defense responses like induced systemic resistance (ISR). ISR is typically characterized by priming of defense responses in distal tissues, enabling plants to respond more rapidly and robustly to subsequent pathogen attacks (Kannojia et al., 2019; He et al., 2024). While the use of PGPM-mediated resistance is proposed as an eco-friendly approach against viral diseases, its effectiveness is often inconsistent. Evidence accumulated over the past two decades has demonstrated that the PGPM-mediated antiviral resistance depends on the specific combination of PGPM strain, plant host, and viral pathogen. For instance, the PGPMs *Pseudomonas fluorescens* 89B-27 and *Serratia marcescens* 90–166 while being able to reduce CMV incidence in cucumber plants, in tomatoes resulted only in a milder symptomatology (Zehnder et al., 2000). Application of *Bacillus amyloliquefaciens* MBI600 in tomato plants resulted in an impressive reduction of TSWV incidence. However, against potato virus Y (PVY, *Potyvirus yituberosi*) only a delay of infection progress was recorded without alteration of the disease index (Beris et al., 2018). On the contrary, *Bacillus subtilis* 1211 EMCCNc resulted in reduced disease severity when used as a biocontrol agent for PVY management in tomato plants (Ghanaim et al., 2025). Taking together all these studies, it is evident that PGPM-induced resistance is not uniformly effective across the distinct virus species.

Virus biology appears to be a critical determinant of this specificity, while PGPM-mediated resistance is further shaped by bacterial traits. In particular, viruses differ substantially in their replication strategies (Hull, 2014), the host mechanisms used for their systemic movement (Boevink and Oparka, 2005), and also their capacity to suppress host defense pathways, such as RNA silencing and hormone-regulated responses (Pumplin and Voinnet, 2013; Alazem and Lin, 2015). The complicated tripartite interactions that are formed among PGPMs, viruses and host plants are mainly orchestrated by phytohormones. It is widely evidenced that colonization of the roots by PGPMs can activate distinct signaling pathways, primarily those involving salicylic acid (SA) and/or abscisic acid (ABA)/ethylene (ET), while jasmonic acid (JA), auxin, gibberellins and brassinosteroids can also demonstrate key roles in fine tuning the induced defense mechanisms (Arkhipova et al., 2007; Pieterse et al., 2014; Glick, 2020; Jiao et al., 2021). On the other hand, viruses belonging to different genera use discrete ways to manipulate the plant hormonal-induced mechanisms in order to promote their infection cycle (Robert-Seilaniantz et al., 2011; Alazem and Lin, 2015). Therefore, the effectiveness of PGPM-induced host resistance pathways depends on their ability to counteract a particular virus infection strategy.

To decipher the molecular basis of ISR in the aerial part of the plants, following PGPM root colonization, numerous transcriptomic studies have been performed either by gene specific analysis or by transcriptome profiling. Overall, it is noted that the transcriptional outcomes of PGPM colonization in the systemic leaf tissues vary. Several ISR-eliciting PGPMs induce little or no transcriptional change in distal leaves and therefore ISR is related to priming, while others can trigger extensive systemic leaf transcriptional reprogramming along with enhanced resistance (Verhagen et al., 2004; Pieterse et al., 2014; Hanifah et al., 2023).

Despite the recent advances, comparative studies examining the effects of a single PGPM strain against multiple viral pathogens within the same host are based mainly to phenotypic analyses (Park et al., 2006; Lee and Ryu, 2016; Beris et al., 2018). Such comparisons are essential to be extended at the molecular level, in order to comprehend why a PGPM-induced host resistant pathway is effective against certain viruses but not to others. Identifying which resistance mechanism is associated for each PGPM-virus-plant combination would allow us to propose the most suitable PGPMs that can efficiently restrict a certain virus threat in a given cultivation. *B. amyloliquefaciens* MBI600 is the active ingredient of the commercially available bio-fungicide Serifel^®^ (BASF, S.E.) and a well-characterized PGPM widely used in agriculture for the enhancement of growth promotion and disease-suppressive properties. Its efficacy against fungal and bacterial pathogens is well documented (Dimopoulou et al., 2019, 2021; Samaras et al., 2022), and while it is known for its antiviral properties against TSWV and PVY, the molecular basis of this virus-specific resistance remains largely unexplored.

Under this context, the aim of the current study was i) to further evaluate the antiviral traits of *B. amyloliquefaciens* MBI600, against TYLCV and CMV, ii) to elucidate the molecular basis of the differential response recorded in the antiviral efficacy of MBI600 against different virus species, and iii) to identify at the systemic level host responses closely associated with the differential antiviral effectiveness of this PGPM. Our findings provide new insights into the selective nature of PGPM-mediated antiviral resistance strengthening the rationality for their deployment as an effective strategy against viral diseases in crops.

## 2 Materials and Methods

### 2.1 Plant material and MBI600 application

The tomato (*Solanum lycopersicum* L.) hybrid ‘Belladonna F1’ was used throughout this study. Plants were grown in an insect-proof greenhouse under controlled environmental conditions. Temperature was set at 25 C/20 °C, (day/night) with recorded extreme values of 28 °C/17 °C (day/night), and photoperiod adjusted to 16 h-light with supplementary to daylight illumination provided by GreenPower LED flowering DR/W and DR/W/FR lamps (22 μmol/s). The experiments were performed over two consecutive years. Plants were grown in soil-less potting medium (Potgrond P, Klasmann) in pots with dimensions 90 mm × 90 mm × 100 mm. No additional fertilization was applied. Serifel^®^ (*B. amyloliquefaciens* MBI600 viable spores) was diluted in sterile water in a concentration of 5.5 x 10^7^ cfu/ml as recommended. 60 mL of the resulting suspension were applied through drenching of plant roots, and a triple drenching program with seven days interval was applied, with the first application of the MBI600 to be at the developmental stage of the second unfolded leaf on main shoot (phenological developmental stage BBCH 102) of tomato plants (Beris et al., 2018).

### 2.2 Virus inoculations and disease assessment

All viral inoculations were performed one day after the second MBI600 application and plants treated with water were used as control. The efficacy of MBI600 was evaluated against CMV-I17F (Beris et al., 2023) and TSWV-619 strains through mechanical inoculation. Briefly, virus inocula were obtained by grinding infected *Nicotiana benthamiana* leaf tissue in 10 mM sodium phosphate buffer pH 7 supplemented with 0.2% w/v DIECA (1:3 w/v dilution). Carborudum (3% w/v) was added to each inoculum, and the resulting mixture was gently rubbed onto one leaflet of the upper fully expanded leaf. The experiments were performed twice and 30 plants per treatment were analyzed. Disease assessment was performed by symptom monitoring., The presence and titre of the viruses were assessed in the apical non-inoculated leaves by double-antibody sandwich enzyme-linked immunosorbent assay (DAS-ELISA) with commercial antibodies specific for each virus (LOEWE Biochemica GmbH, Germany).

For TYLCV, two inoculation methods were used. The first one was based on agro-inoculation of the TYLCV infectious clone (Belabess et al., 2015) kindly provided by Dr. Michel Peterschmitt (French Agricultural Research Centre for International Development, CIRAD, Montpelier, France). For the agro-inoculation of tomato plants the protocol described previously by Jammes et al., 2023 was followed with minor modifications. *Agrobacterium tumefaciens* C58 MP90 strain harboring the TYLCV infectious clone was grown for 48 hours at 28°C with agitation at 150 rpm in LB medium containing 50 μg/mL kanamycin and 30 μg/mL gentamycin. Bacterial cells were collected by centrifugation for 5 min at 4000 rpm and the resulting pellet was resuspended in equal volume of Wash Buffer (WB, 10 mM MgCl_2_, 100 μM acetosyringone). The suspension was then centrifuged again for 5 min at 4000 rpm and the bacterial pellet was resuspended in appropriate amount of Infiltration Medium [¼ MS (Murashige and Skoog medium), 1% sucrose, 0.001% Silwet L-77, 200 μM acetosyringone, pH 6.0] to a final OD_600_ = 1. Approximately 50 μL of the resulting suspension of *A. tumefaciens* was infiltrated with 1-mL needless syringes on the lower epidermis of a leaflet, at the basal space between two leaf veins. In total three leaflets per plant were infiltrated.

The second approach for TYLCV inoculation was based on transmission to tomato plants at the second to third true leaf stage *via* viruliferous whiteflies *Bemisia tabaci* MED. Adult whiteflies were allowed to acquire TYLCV by feeding on infected tomato plants for 24 h. Following the acquisition access period, batches of 50 viruliferous whiteflies were released into two separate insect-proof screen cages, each containing 10 healthy tomato plants. One cage received plants treated with MBI600, while the second cage received water-treated plants. Whiteflies were allowed an inoculation access period of 72 h, after which they were manually removed using an aspirator. The experiment was repeated three times and in total 30 plants per treatment were analyzed. For both agro-inoculation and whitefly transmission, disease progression was assessed by symptom monitoring. TYLCV titer was quantified with qPCR as previously described (Papayiannis et al., 2010). Leaf tissue sampling was performed in two time points and total nucleic acids were extracted from 0.2 g leaf tissue using a guanidine hydrochloride-silica membrane protocol as described by Chatzinasiou et al. (2010). Extracts were treated with DNase-free RNase A (Thermo Fisher Scientific, 10 mg/mL) to remove residual RNA prior to DNA quantification.

Cq values obtained from three biological replicates were analyzed using linear mixed-effects modelling. Treatment (MBI600 vs Water) and time (14 vs 28 dpi) were included as fixed effects, and plant identity was included as a random effect. Pairwise comparisons among treatment-time combinations (MBI600-14, MBI600-28, Water-14, Water-28) were performed using Tukey-adjusted *post-hoc* tests. To assess consistency among biological replicates, differences in mean Cq values were evaluated using one-way ANOVA with replicate as a factor, and homogeneity of variances was tested using Levene’s test. Statistical analyses were performed using R version 4.4.3, with Levene’s test implemented in the *car* package. Differences were considered statistically significant at P < 0.05.

### 2.3 RNA-seq analysis for transcriptome assessment

Transcriptome analysis was conducted using RNA sequencing (RNA-Seq). Tomato plants were grown in a controlled growth chamber (MLR-352H, Panasonic) with 16 h-light photoperiod, light intensity of 20000 lux, temperature 25 °C/22 °C (day/night) and relative humidity 85% (day) and 90% (night). MBI600 application followed the triple drench scheme described previously. Transcriptome was assessed at three timepoints: (i) just prior to virus inoculation (one day after the second drench application), (ii) for CMV and TSWV, at three days post inoculation (dpi) in the upper non-inoculated fully expanded leaf and (iii) for TYLCV, at five days after agro-inoculation in the upper non inoculated fully expanded leaf. Sampling timepoints after virus inoculation were optimized based on the inoculation method used. Three dpi were selected for sap-inoculation of CMV and TSWV as this method provides delivery of intact virions in plant cells ensuring a rapid replication of the virus genome. For TYLCV agro-inoculation, 5 dpi were used as this time window accounts for effective TYLCV genome transfer and expression in cells, and ensures the lack of transient host responses due to the *Agrobacterium* vector (Czosnek et al., 1993; Pitzschke, 2013). For CMV and TSWV, mock inoculated plants (rubbed with phosphate buffer) were included, while for TYLCV, *A. tumefaciens* C58 MP90 strain harboring the empty binary vector was used as control. Three plants were assessed per biological group (12 groups), resulting in a total of 36 samples analyzed by RNA-Seq. Upon sampling, tissues were homogenized with liquid nitrogen and total RNA was extracted from 50 mg of tissue using the RNeasy Plant Mini Kit (QIAGEN GmbH, Hilden, Germany) according to the manufacturer’s instructions. Genomic DNA was removed using DNase I (RNase-free; New England Biolabs, Ipswich, MA, USA) according to the manufacturer’s instructions. RNA sequencing was performed at the Greek Genome Center (GGC), Biomedical Research Foundation of the Academy of Athens (BRFAA, Athens, Greece), on an Illumina NovaSeq 6000 platform (Illumina, San Diego, CA, USA).

### 2.4 RNA-seq data processing

Raw 100 bp single-end RNA-Seq reads were imported into Geneious Prime v 11.1.5 (Geneious; Auckland, New Zealand). Approximately 25 million reads were obtained per sample. Adapter sequences and low-quality bases were removed using the BBDuk plugin with a minimum Phred quality threshold of Q30 and a minimum read length cutoff of 50 bp after trimming. Trimmed reads were then mapped to the reference tomato genome (GCF_036512215.1) using the Geneious RNA mapper with default “medium sensitivity” settings. Mapping performance was evaluated using standard alignment statistics (percentage mapped, coverage distribution, and read depth). Gene-level abundance was quantified by generating a read count table (raw counts per annotated gene/transcript) from the mapping results. Differential expression was performed in Geneious Prime v 11.1.5 using the DESeq2 (Love et al., 2014) plugin. Library size normalization was performed using DESeq2’s median-of-ratios method, which corrects for sequencing depth and RNA composition bias, while dispersion estimates were fit using the default shrinkage procedures. Genes with zero counts across all samples were excluded automatically, while no additional arbitrary low-expression filtering was applied. Log_2_ fold changes (log_2_FC) were estimated, and genes were considered differentially expressed (DEGs) if they met the following thresholds: Benjamini–Hochberg FDR-adjusted p-value (padj) < 0.05 and |log_2_FC| ≥ 1.

### 2.5 Gene ontology annotation and enrichment analysis

Functional annotation of DEGs was conducted using Blast2GO (Conesa et al., 2005). Sequences were queried against the NCBI protein database using BLAST with an E-value cutoff of 1e-5, and GO terms were assigned using the BLAST2GO annotation pipeline (mapping + annotation steps) with default parameters. Annotation outputs were used to generate a GO term association file for downstream enrichment testing. GO enrichment analysis of DEGs was also carried out in Blast2GO using Fisher’s exact test by comparing the set of DEGs (test set) to the set of all expressed genes/transcripts used for differential expression (background/reference set). Multiple testing correction was applied using the Benjamini–Hochberg FDR procedure and GO categories with FDR < 0.05 were considered significantly enriched. Enrichment was examined for the three GO domains: Biological Process, Molecular Function, and Cellular Component.

### 2.6 KEGG pathway enrichment analysis (KOBAS-i)

KEGG pathway annotation and enrichment analysis were performed using KOBAS-i (Bu et al., 2021). FASTA sequences of the DEGs detected were submitted to KOBAS-i for KEGG orthology assignment using the appropriate species reference or the closest available taxon. KEGG enrichment was then assessed using the DEGs as the test set and all expressed genes as the background set. P-values were corrected for multiple comparisons using the Benjamini–Hochberg FDR method, and pathways with FDR < 0.05 were considered significantly enriched.

### 2.7 Verification of RNA-seq results with quantitative RT-PCRassays

To validate the obtained RNA−Seq data, selected differentially expressed genes were analyzed with quantitative reverse transcription PCR (RT−qPCR). For each sample, 1 µg of total RNA used in the RNA-Seq analysis was reverse−transcribed into cDNA using the SMART™ M−MLV Reverse Transcriptase (Takara Bio/Clontech, USA) and oligo(dT) primers according to the manufacturer’s instructions. The resulting cDNA was then diluted with nuclease−free water (dilution 1:5) and used for the subsequent RT-qPCR assays. All assays were performed in a QuantStudio 5 Real−Time PCR System (Applied Biosystems, Thermo Fisher Scientific, USA) with KAPA SYBR^®^ FAST qPCR Master Mix (Roche Diagnostics/Kapa Biosystems, Switzerland) regent according to manufactures instructions. Briefly, all reactions were performed in a final volume of 10 µL containing 1X of KAPA SYBR^®^ FAST qPCR Master Mix, 1X of ROX reference Dye, 0.1 μM of each primer and 1 μL cDNA. The thermal cycling program consisted of an initial denaturation step at 95 °C for 3 min, followed by 40 cycles of denaturation at 95 °C for 3 s and annealing/extension at 60 °C for 30 s. A melt−curve analysis was performed at the end of each run to confirm amplification specificity. Gene expression levels were normalized using *UBIQUITIN3* (*UBI3*) as reference (Rotenberg et al., 2006). Relative expression levels were calculated using the 2^−ΔΔCt^ method and statistical analysis was performed as described in Beris et al., 2018. RT−qPCR results were compared with RNA−Seq data to assess the consistency of expression trends. All primers used are described in **Supplementary Table 1**.

## 3 Results

### 3.1 MBI600 treatment attenuates TYLCV disease symptoms and viral accumulation

**Figure 1:**
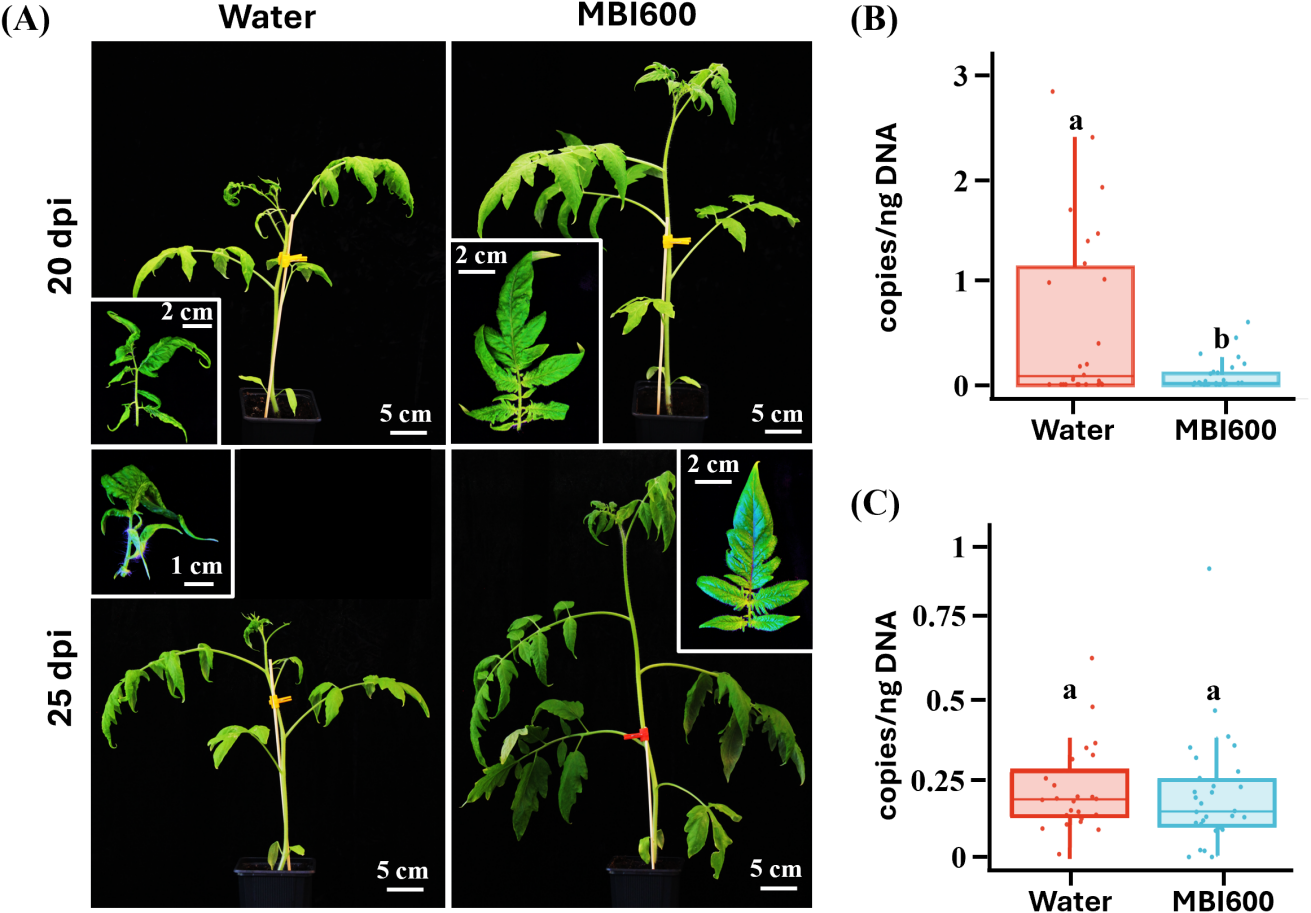
Effect of *Bacillus amyloliquefaciens* strain MBI600 against tomato yellow leaf curl virus (TYLCV) in tomato plants upon agro-inoculation. **(A)** Symptoms observed in water- and MBI600-treated plants at 20 and 25 days post agro-inoculation (dpi). Water-treated plants developed severe symptoms that included, curling yellowing and stunting, while MBI600 remained almost asymptomatic with mild curling and yellowing. Quantitation of virus DNA with qPCR at **(B)** 20 dpi and **(C)** 32 dpi. Different letters indicate statistically significant differences (p<0.05). A significant reduction of TYLCV titer was recorded at 20 dpi.

Since TYLCV is not mechanically transmitted, two inoculation approaches were followed to examine the possible antiviral action of MBI600 against the virus. As demonstrated in **Figures 1A**, symptoms were initially recorded at 20 days post agro-inoculation (dpi). Water-treated control plants displayed typical TYLCV symptoms, including leaf curling and chlorosis. In contrast, MBI600 treatment resulted in reduced symptom severity that remained up to 25 dpi, while, at 32 dpi, approximately 30% of MBI600-treated plants remained symptomless (**Figure 1A**). Quantification of TYLCV DNA by qPCR at 20 dpi showed that the difference observed between MBI600- and water-treated plants in symptom severity was accompanied by a respective statistically significant reduction of TYLCV titer (P = 0.002; **Figure 1B**). However, at 32 dpi the viral DNA levels were comparable between treatments and no significant difference was recorded (P = 0.727, **Figure 1C**).

**Figure 2:**
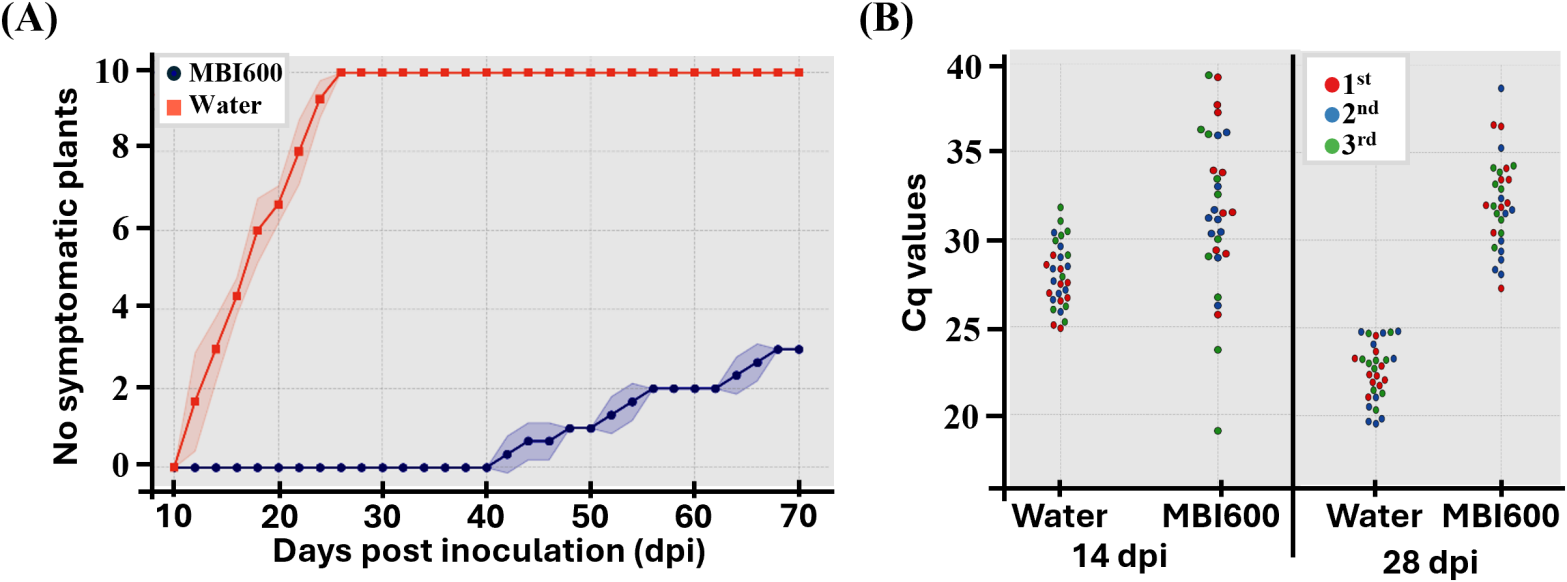
Effect of *Bacillus amyloliquefaciens* strain MBI600 against tomato yellow leaf curl virus (TYLCV) in tomato plants upon transmission with whiteflies, *Bemisia tabaci*, MED. **(A)** Progression of disease symptoms over time in tomato plants treated with either water or MBI600. Each point represents the average number of plants showing TYLCV symptoms. Shaded areas represent the variability among three experiments. **(B)** Swarmplot of Cq values obtained during the quantification of TYLCV with qPCR at 14 and 28 days post transmission (dpi). Higher Cq values indicate lower viral DNA. Each dot represents one plant and different colors represent each experiment. Different letters indicate statistically significant differences (p<0.05).

In accordance with virus agro-inoculation experiments, following transmission by viruliferous whiteflies *Bemisia tabaci* MED, TYLCV disease progression differed between treatments. In water-treated plants, symptom development initiated at 12 dpi and all plants became symptomatic at 26 dpi, while MBI600-treated plants symptoms initially appeared at 42 dpi and throughout the assessment period only 30% of MBI600-treated plants developed symptoms (**Figure 2A**). At 28 dpi, TYLCV accumulation was lower in MBI600-treated plants, as indicated by higher Cq values obtained by qPCR (**Figure 2B**). Cq values were analyzed using a linear mixed-effects model. Statistical analysis revealed significant effects of treatment (χ^2^(1) = 5.05, P = 0.0247) and sampling time [χ^2^(1) = 38.33, P = 5.98 x 10^-10^], as well as a significant treatment x time interaction (χ^2^(1) = 182.79, P < 2.2 x 10^-16^], on Cq values. Pairwise comparisons showed that water-treated plants at 28 dpi exhibited significantly lower Cq values compared with all other treatment-time combinations (Tukey-adjusted *post-hoc* tests, all P< 0.001). In addition, MBI600-treated plants at 28 dpi showed significantly higher Cq values compared with both MBI600- and water-treated plants at 14 dpi. No statistically significant differences were detected between MBI600 and water-treated plants at 14 dpi (ΔCq = 1.65, 95% CI [-0.27 to 3.57], Tukey P = 0.1186). Analysis of biological replicates showed no significant differences in mean Cq values [one-way ANOVA, F(2, 117) = 0.53, P = 0.59] and no significant differences in variance [Levene’s test, F(2, 117) = 0.20, P = 0.82], indicating high consistency among replicates. Finally, both inoculation methods produced comparable results, with consistent trends in symptom development and virus accumulation, among the experimental replications performed.

### 3.2 Effect of MBI600 on CMV infection

In contrast to its effect on TSWV and TYLCV, MBI600 application did not result in significant differences in symptom development, virus incidence, or virus titer in plants infected with CMV. Similarly, virus detection and accumulation assessed by DAS-ELISA did not reveal any significant differences that could be attributed to MBI600 application.

### 3.3 Transcriptomic responses to MBI600 treatment in the presence and absence of viruses

To investigate the molecular basis of the host transcriptional responses associated with MBI600 treatment, RNA-Seq analysis was performed on upper leaf tissues in the absence and presence of viruses and at different timepoints. Consequently, the number of identified DEGs at each time point reflects the combined effects of virus infection kinetics, inoculation method used, and plant defense mechanism induced. In the absence of viral pathogens, no differentially expressed genes (DEGs) were detected in the MBI600-treated plants when compared to water-treated controls at any timepoint analyzed (**Table 1**). This finding indicates that, under the experimental conditions tested, root colonization by MBI600 did not result in transcriptional reprogramming in tomato plants detectable by RNA-Seq.

**Table 1:** Number of differentially expressed genes (DEGs) that were detected in different time points and comparisons.

Timepoint	Virus	Comparisons	No DEGs	Up-regulated	Down-regulated
T0 (prior virus inoculation)	NO	MBI600 vs Water	0	0	0
T1 (3 dpi)	NO	MBI600-Mock vs Water-Mock	0	0	0
	CMV	Water-CMV vs Water-Mock	535	439	96
	CMV	MBI600-CMV vs Water-Mock	418	354	64
	TSWV	Water-TSWV vs Water-Mock	76	18	58
	TSWV	MBI600-TSWV vs Water-Mock	753	598	155
T2 (5 dpi)	NO	MBI600-Agro vs Water-Agro	0	0	0
	TYLCV	Water-TYLCV vs Water-Agro	55	19	36
	TYLCV	MBI600-TYLCV vs Water-Agro	21	15	6

A gene was considered as differentially expressed (DEGs) if it met the following thresholds: Benjamini–Hochberg FDR-adjusted p-value (padj) < 0.05 and |log_2_FC| ≥ 1.

**Figure 3:**
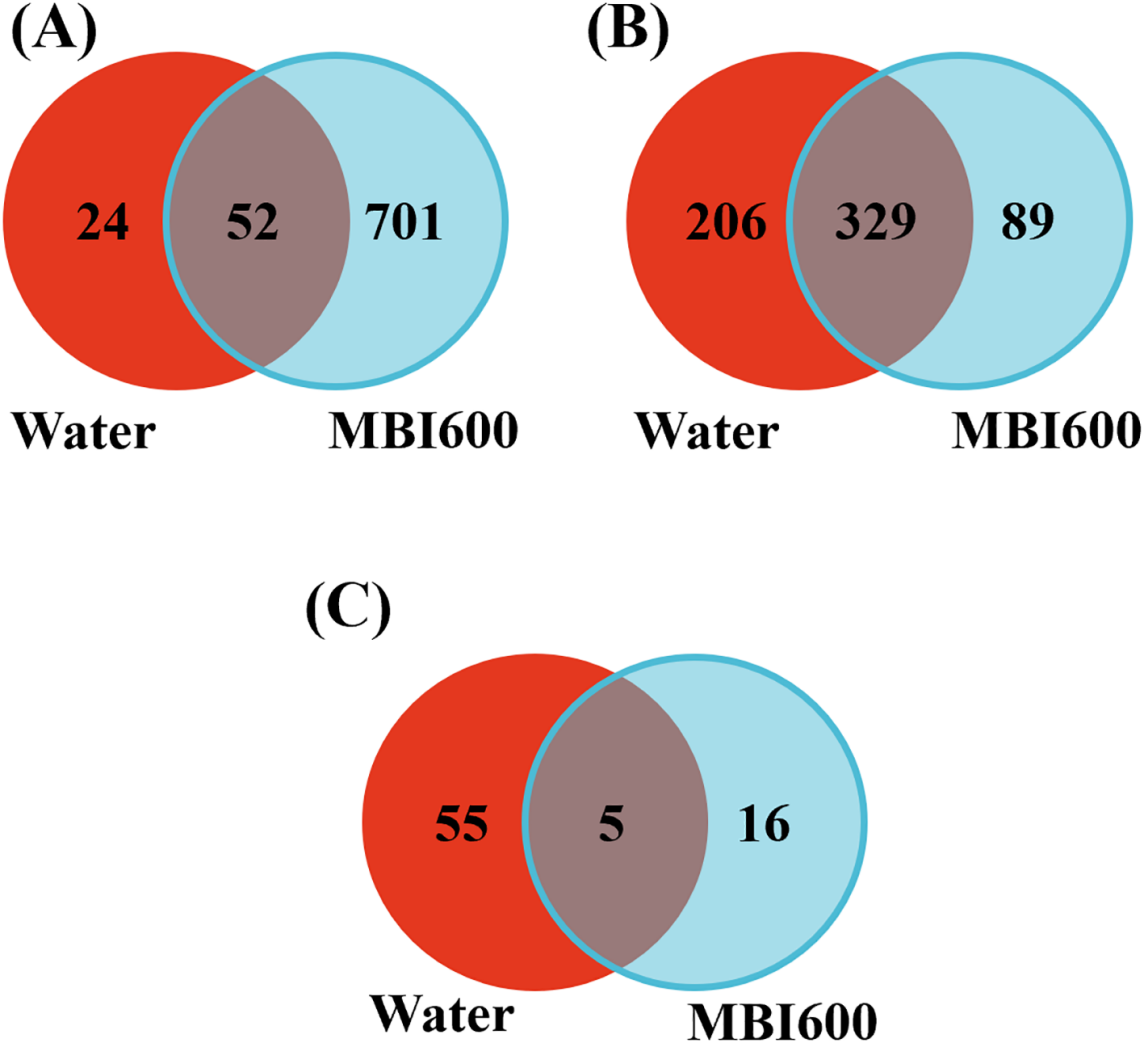
Venn diagrams of the differentially expressed genes (DEGs) identified in water- and MBI600-treated tomato plants upon inoculation with **(A)** tomato spotted wilt virus (TSWV), **(B)** cucumber mosaic virus (CMV) and **(C)** tomato yellow leaf curl virus (TYLCV). DEGs were identified by pairwise comparison of virus-infected plants to their respective mock controls.

In contrast, virus infection triggered distinct transcriptomic responses, that varied depending on the virus and treatment. TSWV infection resulted in a number of DEGs in both water- (76 DEGs) and MBI600-treated plants (753 DEGs), with a substantial notably higher number of DEGs recorded in MBI600-treated plants. CMV infection also induced extensive transcriptional reprogramming changes in host gene expression in the upper non-inoculated tissues. However, comparison of DEG profiles between MBI600- (418 DEGs) and water-treated (535 DEGs) plants revealed a high degree of overlap, with limited treatment-specific differences (**Figure 3B**). The strong similarity in DEG profiles between MBI600- and water-treated plants suggests that CMV infection possibly overrides or bypasses MBI600-induced ISR defense mechanisms. Finally, upon TYLCV inoculation only a small subset of DEGs was recorded in both treatments (55 DEGs in water-treated and 21 for MBI600-treated plants, **Figure 3C**; **Supplementary Table 2**). The limited number of DEGs detected at this early systemic stage probably reflects the restricted TYLCV replication and/or movement at 5 dpi possibly due to the agro-inoculation method followed. Despite the small number of DEGs identified in MBI600-treated plants, the genes which were uniquely detected were associated with oxidative stress mitigation and protein homeostasis. The onset of an MBI600-induced defence mechanism is thus supported and it could be associated with the observed reduced disease phenotype. To assess data reproducibility and overall variance structure, principal component analysis (PCA) was performed using variance-stabilized expression values. Replicates clustered together within each biological group revealing high reproducibility among biological replicates and confirming robustness of the RNA-Seq dataset (**Supplementary Figure 1**). Finally, five DEGs from the RNA-Seq analysis were selected for verification by RT-qPCR. As demonstrated in **Supplementary Table 3** comparable expression trends were obtained between the two methods strengthening the *in silico* results.

### 3.4 Functional enrichment analysis in relation to observed phenotypes

To further contextualize the phenotypic differences observed among treatments, Gene Ontology (GO) and KEGG pathway enrichment analyses were examined in relation to virus-specific disease outcomes. Enrichment testing was conducted using the complete set of DEGs identified for each treatment. All expressed tomato genes/transcripts included in the differential expression analysis were used as the background reference set.

In the presence of viruses, enrichment analysis of GO terms related to *Biological Process* resulted in the identification of four terms (*response to stress*, *regulation of biological process*, *response to stimulus*, and *DNA-templated transcription*) that were overrepresented mostly, but not exclusively, by up-regulated genes. Overexpression occurred to a similar extent both in the presence and absence of the MBI600, and independently of CMV and TSWV. These terms likely reflect the activation of a general antiviral defense and adaptive responses that are commonly induced in plants following viral infection and are not specifically associated with the presence of MBI600.

**Figure 4:**
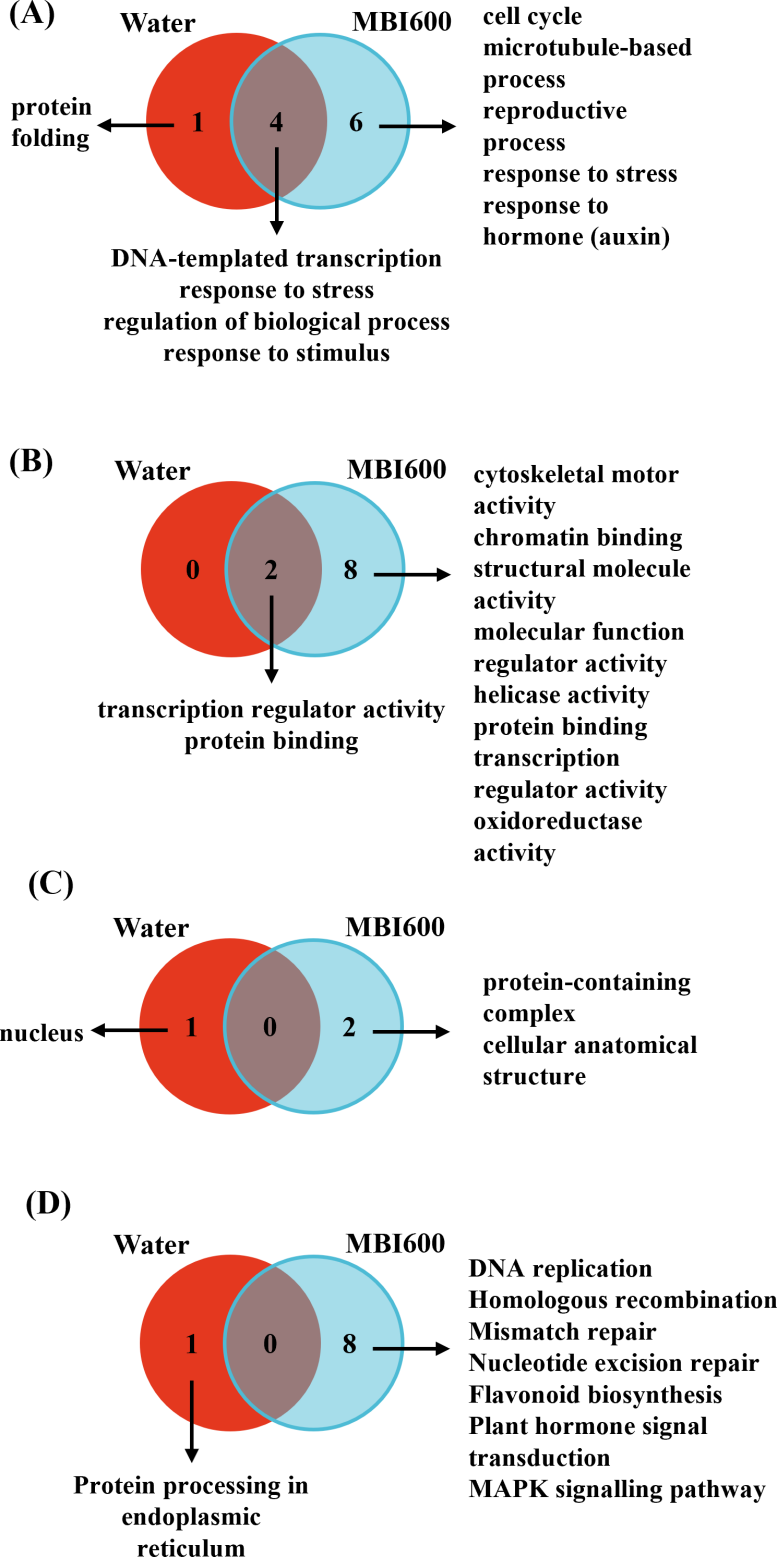
Venn diagrams of enriched gene ontology (GO) terms for **(A)** biological processes (GO:0008150), **(B)** molecular functions (GO:0003674), **(C)** cellular components (GO:0005575) and **(D)** KEGG pathways identified in water- and MBI600-treated tomato plants following TSWV inoculation. Most of the enriched terms identified were specific to MBI600-treated plants, indicating that the transcriptional reprogramming was predominantly induced by MBI600 in response to TSWV infection.

Furthermore, with respect to TSWV inoculation, water-treated control plants exhibited particularly strong enrichment of the *protein folding process*, consisting of down-regulated DEGs encoding chaperone proteins, and indicating a pronounced cellular stress. In contrast, in the presence of MBI600, enrichment was observed for terms related to the *cell cycle* and *cytoskeleton*, along with the activation of hormone-related responses, including *response to auxin* and *to abscisic acid* (**Figure 4A**). Overall, the majority of DEGs identified under MBI600 treatment and associated with enriched GO terms were upregulated. Notably, within the term response to auxin, an approximately 5-fold over-expression of key auxin regulatory genes was observed, including *Auxin Response Factor 5* (*ARF5*), A*uxin efflux carrier component 5* (synonym of *PIN-FORMED 5*, *PIN5*), and A*uxin efflux carrier component 7* (synonym of *PIN-FORMED 7*, *PIN7*). These changes indicate enhanced auxin perception, transport, and signaling specifically in MBI600-treated plants. Auxin signaling has been implicated as an important component of TSWV antiviral defense, as it has been associated with the *Sw-5b* resistance locus conferring resistance against TSWV in tomato (Yang et al., 2025). In contrast, the enriched term *response to abscisic acid* was predominantly composed of down-regulated genes, including the ABA receptor *PYL3* and key ABA biosynthetic enzymes *9-cis-epoxycarotenoid dioxygenase 1* (*NCED1*) and *NCED2*. The down-regulation of these genes suggests a potential attenuation of ABA signaling, which may indirectly favor activation of salicylic acid (SA)-dependent defense mechanisms (Qi et al., 2021).

Additionally, from the respective analysis of GO terms related to *Molecular Function*, MBI600-treatment resulted in up-regulation and overrepresentation of proteins with *oxidoreductase activity*. This suggests the potential modulation of cellular metabolic processes and stress-responsive pathways (**Figure 4B**). Among these DEGs, an 8-fold up-regulation was recorded in the expression of the *Chloroplastic Polyphenol Oxidase B*, associated with programmed cell death and plant defense responses (Newman et al., 2011). Moreover, *cytochrome P450*, which plays a central role in regulating major antiviral defense mechanisms in plants (Abraham et al., 2026), showed a 3-fold overexpression in the MBI600-treated plants. Finally, KEGG analysis revealed the enrichment of additional defense-associated pathways, including flavonoid biosynthesis, MAPK signaling, and plant hormone signal transduction, only in the MBI600-treated samples (**Figure 4D**). Importantly, the flavonoid biosynthetic pathway appears to be of particular significance, as it has been associated with resistance loci against TSWV and is strongly supported here by the overexpression of key biosynthetic enzymes such as *anthocyanidin synthase* (*ANS*), *chalcone synthase 1* (*CHS1*) and *dihydroflavonol 4-reductase* (*DFR*) (Lv et al., 2023). This denotes the induction of secondary defense-related processes during TSWV inoculation in the presence of MBI600 compared to control plants.

**Figure 5:**
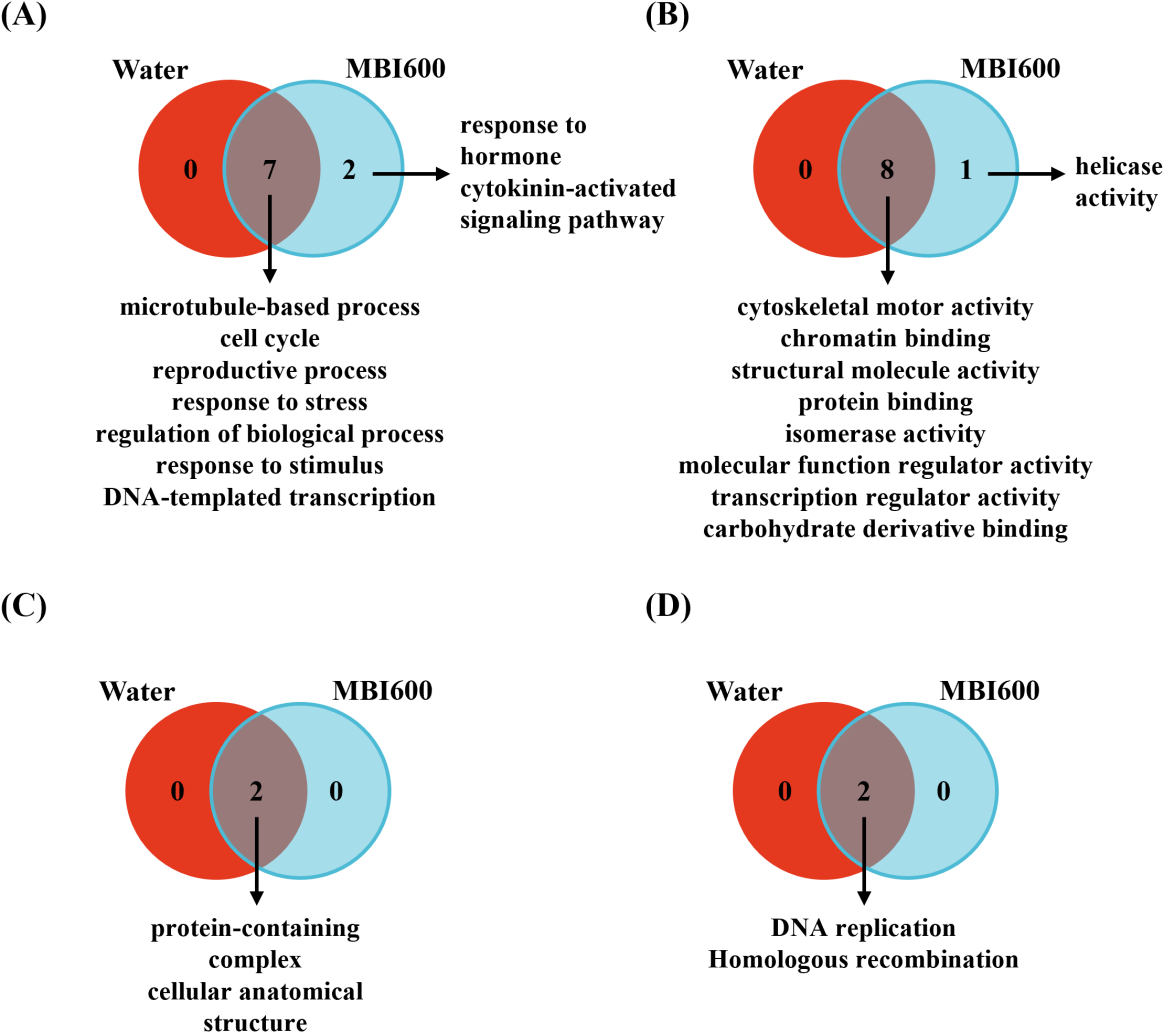
Venn diagrams of enriched gene ontology (GO) terms for **(A)** biological processes (GO:0008150), **(B)** molecular functions (GO:0003674), **(C)** cellular components (GO:0005575) and **(D)** KEGG pathways identified in water- and MBI600-treated tomato plants following CMV inoculation. Most enriched terms were common between the two biological groups reflecting the general CMV-induced transcriptional reprogramming with limited MBI600-specific effect.

Concerning CMV, both in the absence and presence of MBI600, enrichment was observed for GO terms related to the *cell cycle*, *microtubule-based processes*, and *reproductive processes* mostly containing over-expressed genes (**Figure 5A**). These findings highlight the extensive cellular and transcriptional reprogramming as part of the host response to CMV infection. However, in the presence of MBI600, additional enrichment was detected for terms associated with the *cytokinin-activated signaling pathway* and *response to hormone*, suggesting modulation of hormone-dependent mechanisms in PGPM-treated plants during CMV infection, although the latter was not associated with detectable phenotypic differences (**Figure 5**). Regarding the enriched term *response to hormone*, several DEGs were found to be commonly upregulated and to a similar extent in MBI600-treated plants following both CMV and TSWV challenge including *ARF5* and the ABA receptor *PYR1*. Concurrently, two F-box/kelch-repeat proteins, namely F-box/kelch-repeat protein At1g15670 and At1g80440, previously characterized in *Arabidopsis thaliana* as negative regulators of cytokinin signalling (Kim et al., 2013), were down-regulated. In addition, *GATA transcription factor 22* (Köllmer et al., 2011) and *histidine-containing phosphotransfer protein 4* (Hutchison et al., 2006), both components of cytokinin signalling pathway, were upregulated. Collectively, this transcriptional combination strongly supports activation of the cytokinin signaling pathway in MBI600-treated plants during CMV infection.

TYLCV infection was associated with a comparatively limited transcriptional response independently of the treatment. The total number of DEGs detected was low leading to undetectable enriched GO terms and KEGG pathways. However, the small subset of genes uniquely differentially expressed in MBI600-treated plants reveals the presence of genes associated with oxidative stress responses, such as *Alternative Oxidase 1a (AOX1a), Heat Stress Transcription Factor A-7a* (*HsfA7a*), ubiquitin-mediated protein regulation, and several uncharacterized proteins (**Supplementary Table 1**).

Summarizing, the comparison of the responses against the viruses tested revealed both shared and virus-specific transcriptional signatures. General stress-responsive and regulatory pathways were commonly activated upon TSWV and CMV inoculation. Moreover, MBI600 treatment enhanced defense-associated pathways that were particularly pronounced against TSWV, while largely absent during CMV infection. Regarding the effect of MBI600 against TYLCV, despite the limited enrichment, stress-related DEGs were also identified.

## 4 Discussion

PGPMs have been increasingly explored as sustainable tools for crop protection. However, their effects on viral diseases, particularly in relation to virus species-specific infection process and vector-mediated transmission, remain understudied. Following the results of our previous study (Beris et al., 2018), we here investigated the effect of *Bacillus amyloliquefaciens* MBI600, active ingredient of the commercial bio-fungicide Serifel^®^ on virus disease development and host transcriptional responses in tomato plants infected with three genetically distinct viruses. Our results demonstrate that the antiviral activity of MBI600 is virus-dependent, with, in addition to TSWV, pronounced and consistent effects against TYLCV, and undetectable impact on CMV.

Recently, Guo et al. demonstrated the protective effects of *Bacillus amyloliquefaciens* strain Ba13 against vector mediated TYLCV infection (Guo et al., 2019). Ba13 application resulted in reduced TYLCV incidence, symptom severity, and viral accumulation, while these effects were attributed to the induction of systemic resistance and improvements in rhizosphere microecology. In a similar manner, in the current study, MBI600 treatment was also associated with delayed symptom development and reduced TYLCV accumulation, particularly during early stages of infection. Especially under whitefly-mediated transmission, viral accumulation remained consistently low throughout the experiments and approximately 70% of MBI600-treated plants did not develop visible symptoms. In comparison, when an agro-infectious TYLCV clone was used, the effect of MBI600 on TYLCV titer was more transient. Nonetheless, a substantial proportion of MBI600-treated plants (approximately 30%) remained asymptomatic. Therefore, the effectiveness of MBI600 was best recorded under natural vector-mediated virus pressure, than with high-titer artificial inoculations (agro-infiltration). Although in the Ba13 study, the absence of symptoms was related to the absence of TYLCV (Guo et al., 2019), our results suggest that the symptom appearance is not tightly linked to TYLCV presence and even to virus titer. The latter is in line with several studies supporting that the PGPM-induced defense mechanisms can frequently result in improved plant tolerance to viral infections without necessarily suppressing virus replication (Raupach, 1996; Damayanti et al., 2007; Ryu et al., 2007). Indeed, such symptom alleviation is of great agricultural value, as elevated yield losses are often tightly correlated to severe symptoms rather than with virus load (Gao and Lozano-Durán, 2025).

The molecular basis of Ba13 enhanced resistance was studied by transcriptomic analyses at 14 dpi. It was attributed to the induction of the RNA interference silencing mechanism that included the altered expression pattern of *ARGONAUTE* gene family and increase of viral genome methylation (Guo et al., 2023). In contrast, our results concerning the transcriptomic responses to TYLCV in distal tissues of MBI600-treated plants were limited and did not reveal broad enrichment of the RNAi mechanism. The limited number of DEGs identified upon TYLCV agro-inoculation could be attributed firstly to the sampling time point (5 dpi) selected for the transcriptome analysis, in order to ensure sufficient viral establishment while minimizing agrobacterium-induced defense host responses (Czosnek et al., 1993; Pitzschke, 2013). Secondly, limited number of DEGs may be attributed to a delayed TYLCV replication and movement due to the agro-infiltration delivery into the plants, compared to the RNA viruses that were sap inoculated. Nonetheless, our analysis has not only captured the onset of systemic host defense responses against the virus, but it was also possible to distinguish the MBI600-specific responses during TYLCV infection. These were primarily linked to stress mitigation and protein homeostasis and provide valuable insights into the putative MB600-induced mechanisms against TYLCV forming a strong basis for future research. Taken together, these findings verify that while different *B. amyloliquefaciens* strains can lead to common resistance phenotypic outcomes, e.g. delay in disease development, the underlying molecular mechanisms may substantially vary. Moreover, further transcriptomic analyses in the presence of the whitefly vector, along with comparisons among the respective transcriptome profiles, could provide deeper insights into the tolerance recorded in this pathosystem (Serteyn et al., 2020), as whitefly-mediated transmission might also be influenced by MBI600 treatment and could affect both plant and vector responses.

Plant viruses are also known to exploit host hormone signaling to promote replication, movement, and symptom development, while effective antiviral defenses rely on balanced hormonal crosstalk and tight regulatory control (Alazem and Lin, 2015). In particular. salicylic acid (SA) is considered as the key antiviral hormone that mainly interferes with virus replication, cell-to-cell and systemic movement, as well as symptom development (Robert-Seilaniantz et al., 2011; Murphy et al., 2020). SA is also known as an inseparable part of hypersensitive reaction and systemic acquired resistance (SAR) (Enyedi et al., 1992; Lawton et al., 1995). In contrast, jasmonic acid (JA) and ethylene (ET) frequently antagonize SA-dependent defenses and are manipulated by viruses to modulate host immunity and cellular susceptibility. Furthermore, auxin signaling is commonly influenced during virus infection resulting in cell cycle reprogramming and tissue permissiveness, thereby supporting viral replication and systemic spread (Alazem and Lin, 2015). Abscisic acid (ABA) also contributes to virus–host interactions by regulating stress responses and plasmodesmatal permeability, influencing viral movement while also modulating immune signaling (Alazem and Lin, 2017). Lastly, cytokinin signaling interacts with salicylic acid–mediated immune pathways and has been implicated in priming and amplifying defense responses, suggesting a role in shaping the outcome of virus infection (Choi et al., 2011; Argueso et al., 2012). The GO term analysis obtained from our transcriptomic analysis showed that the GO term *response to hormone* was enriched in the MBI600 treated plants and was independent of the virus. The latter indicates that MBI600 treatment results in a hormonal reprogramming upon virus inoculation suggesting the involvement of hormones in the MBI600-induced mechanism. By adjusting hormonal signaling networks, PGPM-induced ISR can enhance the speed and intensity of plant immune responses upon virus challenge, often resulting in more rapid expression of defense-related genes and metabolites when the plant is exposed to biotic stress (Pieterse et al., 2014).

Unlike the well-documented antiviral effects of several other PGPMs (Zehnder et al., 2000; Ryu et al., 2004; Dashti et al., 2012), MBI600 treatment did not influence CMV symptom development, disease incidence, or viral accumulation. The inability of MBI600 to induce an antiviral effect against CMV is in line with the transcriptomic analysis and the lack of identification of detectable changes by RNA-Seq. CMV infection induced extensive transcriptional changes with enrichment patterns being largely similar between MBI600 and water treatments, with the involvement of cell cycle and microtubule-based processes and stress related responses. Although analysis of MBI600-treated plants resulted in the additional enrichment of cytokinin-related signaling and hormone response terms, these transcriptional differences were not translated into measurable phenotypic effects, underlying the complexity of hormone-mediated regulation during viral infection (Alazem and Lin, 2015). In the Arabidopsis-CMV pathosystem, cytokinin metabolism is altered resulting in reduced cytokinin levels and reformed root morphology (Vitti et al., 2013). Therefore, cytokinins (CKs) may play an essential role in antiviral defense possibly through modulation of salicylic acid (SA)-induced pathways. Although this term was enriched in MBI600-treated plants, no protective effect against CMV was observed. The lack of efficacy of MBI600 could be thus due to the properties of the infection strategy of this virus, since CMV is a rapidly replicating RNA virus that encodes a strong suppressor of RNA silencing. More specifically, the CMV 2b silencing suppressor not only disrupts the RNA silencing machinery by directly interacting with AGO1 (Zhang et al., 2006), but also plays a key role in overcoming SA-induced antiviral resistance and modulates the accumulation of SA during infection (Zhou et al., 2014). Thus, despite an activation of SA-mediated antiviral mechanism by MBI600 treatment via cytokinin signaling, CMV may circumvent it through the multifunctional activities of its 2b protein. Taken together, these findings further support that PGPM-mediated antiviral resistance is not uniform and must be evaluated on a virus-specific basis highlighting the importance of each virus particular biology.

**Figure 6:**
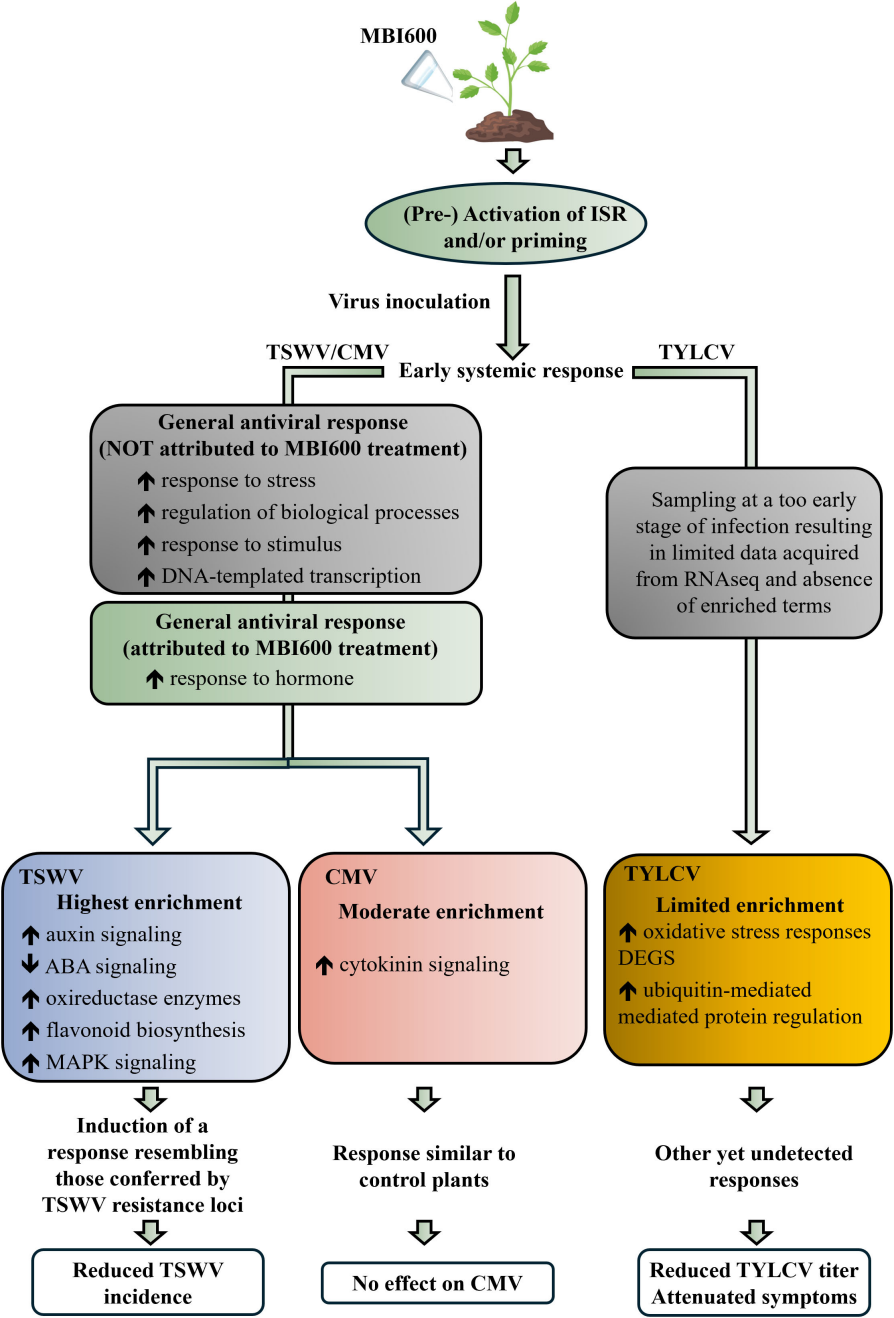
Conceptual model of the differential MBI600-mediated transcriptional response against viruses tested. Schematic representation of the effect of MBI600 root colonization on plant immune responses following viral infection. General antiviral responses correspond to terms enriched in both MBI600- and water-treated control plants.

So far the most promising antiviral activity of MBI600, is recorded against TSWV, as it resulted in a significant reduction of virus incidence (Beris et al., 2018). In this study, we aimed to unravel the molecular basis of this effect by applying RNA-Seq and assessing MBI600-induced transcriptome. The analysis demonstrated extensive transcriptional reprogramming in MBI600-treated plants compared to water-treated ones. Enrichment of cell cycle and cytoskeleton-related processes, hormone-dependent pathways, and secondary defense-associated KEGG pathways such as MAPK signaling and flavonoid biosynthesis was evidenced. More importantly, the auxin response pathway was uniquely enriched only in MBI600-treated plants. Although auxin is considered as a negative regulator of antiviral defense, in the tomato-TSWV pathosystem, enrichment of auxin-related pathways were linked, among others, with the genetic *Sw7* tolerance locus (Padmanabhan et al., 2019). *Sw-7* is a quantitative resistance locus that confers partial but durable resistance to TSWV, and, although not fully characterized, it is tightly associated with hormone reprogramming and robust defense responses during the early infection stages (Padmanabhan et al., 2019). Furthermore, solely in MBI600-treated plants an enrichment in flavonoid biosynthetic pathway was recorded. Notably, resistance to TSWV in the YNAU335 inbred tomato line has been associated with *CHALCONE SYNTHASE 3*, a key enzyme initiating flavonoid production, suggesting a potential role for flavonoid biosynthesis in antiviral defense (Lv et al., 2022). Under this context, MBI600 treatment could lead to similar defense responses and transcriptional reprogramming that could lead to the observed enhanced antiviral effect and the reduction of TSWV incidence. Collectively, a conceptual model of the differential MBI600 effect and mediated transcriptional responses against the three viruses tested is illustrated in **Figure 6**.

Lastly, priming is defined as “a physiological state that enables plants to respond to biotic or abiotic stress more rapidly and/or strongly than non-primed plants upon challenge”, and is a well-established feature of the ISR mechanism mediated by PGPMs (Conrath et al., 2006). In our study, the absence of constitutive activation of defense- or stress-related genes in MBI600-treated plants, across three distinct time points and in the absence of viral infection, suggests that the MBI600-induced defense response is based on a priming mechanism. This interpretation is further supported by the transcriptomic differences observed between water- and MBI600-treated plants following TSWV inoculation. The substantially higher number of DEGs recorded in MBI600-treated plants indicates a faster and more robust transcriptional response, which is a hallmark of priming. In support, a previous work by Samaras and co-workers have demonstrated that MBI600 produces surfactin and fengycin, two anti-microbial substances known to function as priming elicitors in plants (Samaras et al., 2021). This priming-based mode of action is particularly advantageous for agricultural use, as constitutive defense activation can impose fitness costs and negatively affect plant growth (Heil and Baldwin, 2002). However, functional validation experiments were not conducted in the present study. Therefore, although the transcriptomic data strongly suggest the activation of a priming-based ISR following MBI600 treatment, further experimental investigation is required to functionally characterize the proposed priming mechanism.

Overall, this study constitutes one of the first attempts to evaluate the efficacy of a single PGPM, *B. amyloliquefaciens* MBI600, against a range of genetically distant viruses and to characterize the molecular basis of the complicated interactions among host plants, viruses and PGPMs. By combining phenotypic evidence with transcriptomic and functional enrichment analyses, this work strengthens the molecular basis of the observed protective effects of MBI600 and highlights its potential role in integrated management strategies against viruses. Collectively, this integrative approach supports the concept that the efficacy of MBI600-induced resistance depends on the compatibility between the host signaling networks induced and the biological properties of the respective virus. The consistency between studies underscores the robustness of MBI600-mediated anti-virus specific effects and supports its further evaluation under field conditions.

## Data Availability

The data presented in the study are deposited in the NCBI GenBank repository (https://www.ncbi.nlm.nih.gov/genbank/), accession number PRJNA1400300.
