# Supplementary material for: Elucidating the differential antiviral action of a plant growth promoting rhizobacterium against three genetically distant virus species
Source: Front Plant Sci. 2026 Mar 13;17:1778459. doi: 10.3389/fpls.2026.1778459 (PMC13021824; doi:10.3389/fpls.2026.1778459)
Supplement: Supplementary file 1 [file DataSheet1.docx]

Supplementary Material

Supplementary Table 1. Primers used in this study for the verification of the RNA-Seq results.

| **Primer Name** | **Sequence (5'-3')** | **Transcript ID** |
| --- | --- | --- |
| UBI3-F | GCCGACTACAACATCCAGAAGG | NM_001346406.1 |
| UBI3-R | TGCAACACAGCGAGCTTAACC |  |
| ANS-F | GGGTTGGGATTGGAGGAAGG | NM_001374394.1 |
| ANS-R | TCTGGTTGGGGACATTTGGG |  |
| CHS1-F | CGCTAAGCTCCTAGGGCTTC | NM_001247104.2 |
| CHS1-R | CAAGTCCTTGGCTAGCCGAA |  |
| DFR-F | GGACTTGCCCGTAGTGTCTT | NM_001247479 |
| DFR-R | TTGCTTCTGTCGGCAAGTCT |  |
| NCED2-F | GGGTTGAATCAGCCGAAACG | NM_001375893 |
| NCED2-R | TCTGGTGGTGTCATGCATGA |  |
| PYR1-F | GACGGTGCATGGATTCGAAC | XM_004245845 |
| PYR1-F | AAGGCGTGTATCTTCCTCCG |  |

Supplementary Table 2. Differentially expressed genes (DEGs) uniquely identified in MBI600-treated plants upon TYLCV inoculation. In the case of uncharacterized genes, the predicted amino acid sequences were used for an InterProScan and AlphaFold search.

| Transcript id | Description | Expression | Associated GO terms | InterProScan/Alpha-fold |
| --- | --- | --- | --- | --- |
| NM_001247188.2 | alternative oxidase 1a | UP | P:alternative respiration; F:alternative oxidase activity; F:metal ion binding; F:ubiquinol:oxygen oxidoreductase activity; F:superoxide-generating NADPH oxidase activity; C:mitochondrial inner membrane; C:respiratory chain complex |  |
| NM_001247254.1 | plastid lipid associated protein CHRC | UP | P:response to water deprivation; C:chloroplast thylakoid membrane |  |
| XM_004230409.5 | glyoxylase I 4 | DOWN | F:lactoylglutathione lyase activity; F:metal ion binding |  |
| XM_004231990.5 | uncharacterized protein | DOWN |  | None predicted / Ovate family protein (average pLDDT 57.91 - Low) |
| XM_004232835.4 | uncharacterized protein | DOWN |  | PDDEXK-like protein family of unknown function / DUF506 domain-containing protein (average pLDDT 71.5 - High) |
| XM_004234322.5 | CEN-like protein 1 | UP | P:flower development; P:vegetative to reproductive phase transition of meristem; C:cytoplasm |  |
| XM_004237949.5 | uncharacterized protein | UP |  | None predicted / None predicted |
| XM_004238775.5 | E3 ubiquitin-protein ligase MPSR1 | UP | P:protein polyubiquitination; P:protein autoubiquitination; P:regulation of protein complex stability; P:cytoplasm protein quality control by the ubiquitin-proteasome system; F:zinc ion binding; F:misfolded protein binding; F:ubiquitin protein ligase activity; C:cytosol; C:membrane |  |
| XM_004243387.4 | uncharacterized protein | UP |  | None predicted / None predicted |
| XM_004245755.5 | nudix hydrolase 8-like | UP | F:NADH pyrophosphatase activity; F:ADP-ribose diphosphatase activity; F:NAD binding |  |
| XM_004249578.5 | short-chain dehydrogenase TIC 32 B, chloroplastic | UP |  |  |
| XM_010318418.4 | serine carboxypeptidase-like 31 | DOWN | P:proteolysis; F:serine-type carboxypeptidase activity; C:extracellular region; C:membrane |  |
| XM_010320157.4 | dnaJ homolog subfamily B member 6 isoform X2 | UP | F:molecular_function; C:cellular_component |  |
| XM_010328119.3 | heat stress transcription factor A-6b isoform X8 | UP |  |  |
| XR_011220503.1 | PREDICTED: uncharacterized protein LOC102589632 | UP |  | Long non-coding RNA (lncRNA) |
| XR_743281.4 | heat stress transcription factor A-7a isoform X6 | UP |  |  |

P: biological process, F: molecular function, C: cellular component

UP: Up-regulation, DOWN: down-regulation

Supplementary Table 3. Comparison of the log_2_FC and P values obtained by RT-qPCR assays with those obtained during the RNA-Seq analysis. For the RNA-Seq analysis the False Discovery Rate (FDR) is presented. For the statistical analysis of the RT-qPCR results a mixed-linear-model was used as described in Beris et al., 2018. Degrees of freedom were calculated using the Kenward–Roger method and results are presented with 95% confidence intervals.

| **Transcript ID** | **Description** | **Treatment** | **qPCR** | | **RNA-Seq** | |
| --- | --- | --- | --- | --- | --- | --- |
|  |  |  | **Log_2_FC** | **P value** | **Log_2_FC** | **FDR value** |
| NM_001374394 | anthocyanidin synthase | Mock_MBI600 | -0.59329 | 0.200218 | 0.087793 | 0.99988 |
|  |  | TSWV_MBI600 | 1.991303 | 0.000127 | 1.927665 | 2.72E-07 |
|  |  | TSWV_Water | 0.519721 | 0.260303 | 0.374538 | 0.78 |
| NM_001247104 | chalcone synthase 1 | Mock_MBI600 | 0.405 | 0.3982 | 0.172771 | 0.99988 |
|  |  | TSWV_MBI600 | 1.53 | 0.003 | 1.626083 | 1.39E-09 |
|  |  | TSWV_Water | 0.736 | 0.1302 | 0.661387 | 0.42 |
| NM_001247479 | dihydroflavonol 4-reductase | Mock_MBI600 | -0.405 | 0.4474 | 0.066845 | 0.99988 |
|  |  | TSWV_MBI600 | 2.035 | 0.0005 | 1.621936 | 3.57E-05 |
|  |  | TSWV_Water | 0.75 | 0.1641 | 0.641694 | 0.511 |
| NM_001375893 | 9-cis-epoxycarotenoid dioxygenase 2 | Mock_MBI600 | 0.214 | 0.5696 | -0.13519 | 0.99988 |
|  |  | TSWV_MBI600 | -1.66 | 0.0001 | -1.48334 | 0.000101 |
|  |  | TSWV_Water | -0.276 | 0.4624 | -0.14859 | 0.96 |
| XM_004245845 | abscisic acid receptor PYR1 | Mock_MBI600 | 1.027 | <.0001 | 0.804343 | 0.017511 |
|  |  | TSWV_MBI600 | 1.136 | <.0001 | 0.68486 | 0.075324 |
|  |  | TSWV_Water | 0.338 | 0.17 | 0.137718 | 0.99988 |
|  |  | CMV_MBI600 | 1.219 | <.0001 | 1.300735 | 1.87E-05 |
|  |  | CMV_Water | 0.84 | <.0001 | 0.72723 | 1.64E-02 |

Supplementary Table 4. Enrichment scores and False Discovery Rate (FDR) values of the GO terms and KEGG pathways found enriched in the different biological groups.

| **GO terms / KEGG pathways** | | **TSWV-Water** | | **TSWV-MBI600** | | **CMV-Water** | | **CMV-MBI600** | |
| --- | --- | --- | --- | --- | --- | --- | --- | --- | --- |
|  |  | **Enrichment Score** | **FDR** | **Enrichment Score** | **FDR** | **Enrichment Score** | **FDR** | **Enrichment Score** | **FDR** |
| Biological Process | DNA-templated transcription | 2.5 | 1.80E-04 | 0.9 | 1.50E-04 | 0.8 | 2.40E-02 | 1.0 | 5.00E-02 |
|  | cell cycle | ND | | 12.3 | 5.7E-70 | 12.8 | 9.90E-55 | 13.6 | 6.50E-48 |
|  | cytokinin-activated signaling pathway | ND | | ND | | ND | | 1.0 | 4.00E-02 |
|  | microtubule-based process | ND | | 10.2 | 3E-23 | 13.4 | 7.00E-25 | 14.6 | 2.10E-22 |
|  | protein folding | 15.9 | 1.40E-04 | ND | | ND | | ND | |
|  | regulation of biological process | 2.0 | 1.20E-06 | 1.1 | 2.4E-17 | 1.0 | 1.50E-09 | 1.4 | 9.90E-08 |
|  | reproductive process | ND | | 1.8 | 1.10E-08 | 2.0 | 1.80E-07 | 10.5 | 1.40E-06 |
|  | response to auxin | ND | | 2.6 | 3.90E-03 | ND | | ND | |
|  | response to hormone | ND | | 1.4 | 1.10E-04 | ND | | 0.9 | 3.10E-02 |
|  | response to stimulus | 1.9 | 2.80E-06 | 1.4 | 1.6E-26 | 0.9 | 4.30E-08 | 1.4 | 3.00E-08 |
|  | response to stress | 2.1 | 5.20E-04 | 1.7 | 3.5E-22 | 1.2 | 3.30E-08 | 2.2 | 4.90E-08 |
| Molecular Function | cytoskeletal motor activity | ND | | 14.3 | 1.60E-13 | 18.0 | 1.70E-13 | 21.7 | 5.60E-14 |
|  | chromatin binding | ND | | 3.8 | 5.30E-05 | 3.5 | 1.30E-02 | 5.2 | 2.30E-04 |
|  | structural molecule activity | ND | | 2.9 | 7.00E-07 | 2.4 | 3.40E-03 | 3.9 | 5.40E-06 |
|  | protein binding | 1.8 | 1.40E-02 | 2.3 | 4.00E-48 | 2.3 | 1.50E-33 | 2.5 | 7.30E-30 |
|  | isomerase activity | ND | | 2.6 | 6.40E-08 | 2.3 | 3.90E-04 | 4.0 | 2.40E-09 |
|  | molecular function regulator activity | ND | | 2.4 | 2.60E-07 | 2.0 | 3.70E-03 | 2.8 | 7.10E-05 |
|  | transcription regulator activity | 3.6 | 7.20E-02 | 1.3 | 3.40E-05 | 1.0 | 8.60E-02 | 1.0 | 2.90E-02 |
|  | carbohydrate derivative binding | ND | | 0.9 | 2.50E-05 | 0.9 | 7.80E-04 | 1.3 | 1.10E-06 |
|  | helicase activity | ND | | 2.4 | 5.00E-03 | ND | | 3.6 | 3.50E-02 |
|  | oxidoreductase activity | ND | | 1.0 | 8.40E-03 | ND | | ND | |
| Cellular Component | cellular anatomical structure | ND | | 1.2 | 1.30E-11 | 1.0 | 2.50E-05 | 1.3 | 1.50E-06 |
|  | cellular anatomical structure | ND | | 0.7 | 1.10E-43 | 0.7 | 9.20E-30 | 0.6 | 4.50E-20 |
|  | nucleus | 1.8 | 7.20E-03 | 1.7 | 9.00E-46 | 1.6 | 1.90E-26 | 2.1 | 2.30E-34 |
| KEGG Pathways | Homologous recombination | ND | | 17.6 | 3.02E-08 | 14.7 | 3.05E-04 | 23.6 | 1.25E-07 |
|  | DNA replication | ND | | 32.4 | 1.63E-15 | 14.7 | 1.76E-03 | 33.4 | 1.74E-09 |
|  | Protein processing in endoplasmic reticulum | 16.4 | 6.09E-03 | ND | | ND | | ND | |
|  | Flavonoid biosynthesis | ND | | 8.9 | 9.02E-04 | ND | | ND | |
|  | MAPK signaling pathway - plant | ND | | 3.7 | 2.47E-03 | ND | | ND | |
|  | Plant hormone signal transduction | ND | | 3.5 | 9.75E-03 | ND | | ND | |

ND: Not detected.


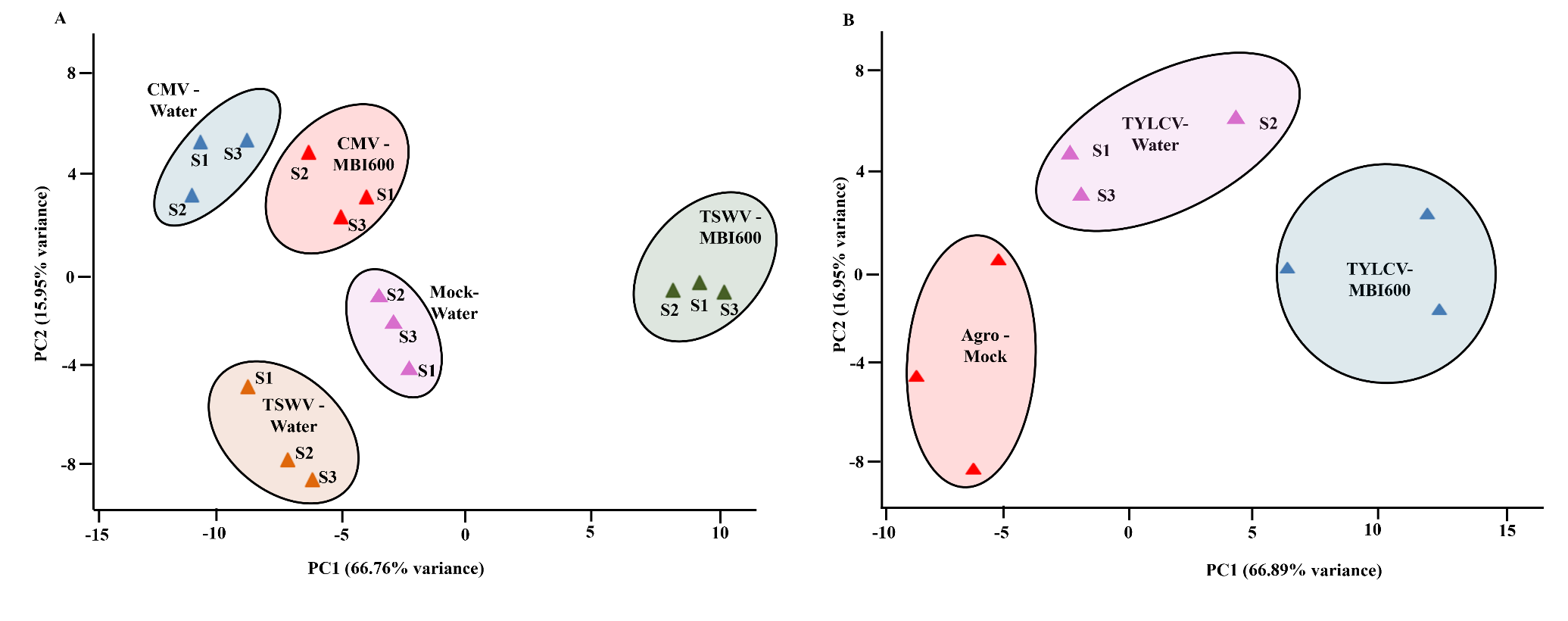


Supplementary Figure 1. (A) Principal component analysis (PCA) of RNA-Seq samples at 3 dpi. PCA was performed using normalized gene expression values from mock-, CMV- and TSWV-inoculated tomato plants treated with either water or MBI600. PC1 and PC2 explain 66.76% and 15.95% of the total variance, respectively. (B) PCA plot of RNA-Seq samples at 5 dpi. PCA was performed using normalized gene expression values from mock-Agro and TYLCV-inoculated tomato plants treated with either water or MBI600. PC1 and PC2 explain 66.89% and 16.95% of the total variance, respectively. In both cases replicates of each biological group cluster closely indicating high reproducibility and consistency of the RNA-Seq dataset.
